# Effects of Dietary Supplementation of Lauric Acid on Lactation Function, Mammary Gland Development, and Serum Lipid Metabolites in Lactating Mice

**DOI:** 10.3390/ani10030529

**Published:** 2020-03-22

**Authors:** Lin Yang, Qiang Yang, Fan Li, Wuzhou Yi, Fangfang Liu, Songbo Wang, Qingyan Jiang

**Affiliations:** 1 Guangdong Provincial Key Laboratory of Animal Nutrition Control, College of Animal Science, South China Agricultural University, Wushan Road No.483, Guangzhou 510642, Guangdong, China; yanglinlover@163.com (L.Y.); yq395586140@163.com (Q.Y.); kyanzedd@163.com (F.L.); lys18639375538@163.com (F.L.); songbowang@gmail.com (S.W.); 2 National Engineering Research Center for Breeding Swine Industry and UBT Lipid Suite Functional Fatty Acids Research Center, South China Agricultural University, Wushan Road No.483, Guangzhou 510642, Guangdong, China; 3 School of Pharmacy and Food Science, Zhuhai College of Jilin University, Caotang, Zhuhai 519041, Guangdong, China

**Keywords:** lauric acid, lactating mice, mammary gland, lipidomics

## Abstract

**Simple Summary:**

Milk secreted from mammary glands is an important nutrition source for offspring after parturition. Mammary gland development and lactation ability have important effects on the growth and health of the offspring. Many studies have demonstrated that external factors, including the environment and nutrition influence the development of mammary glands. Lauric acid is a fatty acid that has many nutritional and physiological properties. In this study, we investigated the effects of dietary supplementation of lauric acid on lactation function and mammary gland development in lactating mice. We found that dietary supplementation of lauric acid during lactation might enhance the mammary development to promote the lactation function of mice. Through the study of mice, we hoped that the results could be applied to animal feed development and animal breeding production.

**Abstract:**

Our previous studies demonstrated that lauric acid (LA) stimulated mammary gland development during puberty. However, the roles of LA on lactation in mice remain indeterminate. Thus, the aim of this study was to investigate the effects of dietary LA supplementation on lactation functioning and to study the potential mechanisms during lactation. in vivo, there was no effect of 1% LA dietary supplementation during lactation on the feed intake or body weight of breast-feeding mice. However, maternal LA supplementation significantly expanded the number of mammary gland alveoli of mice during lactation and the average body weight of the offspring, suggesting that LA supplementation enhanced the development and lactation function of the mammary glands. in vitro, 100 μM of LA significantly increased the content of triglycerides (TG) in the cell supernatant of induced HC11 cells, however, with no effect on the expression of the genes associated with fatty acid synthesis. LA also activated the phosphatidylinositol 3-kinase (PI3K)/protein kinase B (Akt) signaling pathway. LA dietary supplementation significantly expanded the serum levels of lipid metabolites, including sphingomyelin and other metabolites with the sn-2 position of C12 and sn-1 position of C18 in the TG of the lactating mice. Taken together, dietary supplementation of LA during lactation could promote the lactation function of mice, which might be related to increasing the development of the mammary glands and alternation of serum lipid metabolites. These findings provided more theoretical and experimental basis for the application of lauric acid in the development of mammary glands and lactation function of lactating animals.

## 1. Introduction

The mammary gland is an important tissue for the lactation and feeding of offspring, and distinguishes mammals from all other animals [1,2,3]. Mammary gland development involves three major stages, including embryonic, pubertal, and reproductive-cycle stages, which involve pregnancy, involution, and lactation-induced periodic changes [4]. Milk secreted by the mammary gland is an important source of nutrition for the offspring after parturition; thus, mammary gland development and lactation ability have important effects on the growth and health of offspring [5]. Therefore, nutritional regulation to promote mammary gland development may greatly benefit offspring.

Environmental factors [6,7,8,9], hormones [2,10,11,12,13], and nutrition can influence mammary gland development. Numerous studies have shown that nutrition has important effects on mammary gland development during puberty [14], pregnancy [15], and lactation [16,17]. Hormones including prolactin (PRL), growth hormone, and progesterone can regulate the development of mammary glands during lactation. The process of the alveolus developing into milk-secreting lobules is cooperatively regulated by PRL and progesterone [2]. Our previous studies demonstrated that dietary supplementation with 1% lauric acid (LA) increased the expansion of mice mammary ductal during puberty, and 100 μM LA promoted the proliferation in HC11 mouse mammary epithelial cells [14,18]. LA is a medium-chain fatty acid composed of 12 carbons and can be isolated from coconut oil and palm kernel oil [19]. Studies have shown that about two-thirds of the LA in coconut oil are transported via the portal vein and one-third is brought to the lymph and stored in chylomicrons [19,20]. LA is a saturated fatty acid, and its unique fatty-acid properties were shown to have various beneficial effects on health [21].

This study aimed to investigate the effects of LA on the lactation function in mice. We also explored the underlying mechanism of this process, including the relevant signaling pathway in cell, and we found that LA may stimulate the development of mammary glands in lactating mice by activating the phosphatidylinositol 3-kinase (PI3K)/ protein kinase B (Akt) signaling pathway along with altering the lipid metabolism.

## 2. Materials and Methods

All animal experiments were accepted by the Animal Ethics Committee of South China Agricultural University, with permit number SYXK (Guangdong) 2014-0136. The principles for the care and use of animals authorized by the Animal Ethics Committee of South China Agricultural University were in accordance with all animal care procedures that were performed. C57BL/6J mice were bought from Guangdong Medical Laboratory Animal Center (Foshan, Guangdong, China). HC11 mouse mammary epithelial cells were obtained from Shanghai Institutes for Biological Sciences, Chinese Academy of Sciences (Shanghai, China).

### 2.1. Reagents and Antibodies

LA, epidermal growth factor (EGF), and dexamethasone involved in the in vitro experiments were bought from Sigma-Aldrich (St. Louis, MO, USA). The LA used in the in vivo analysis was bought from Chengdu Chemical Technology (Chengdu, China). The PI3K/Akt inhibitor wortmannin (WT) was bought from Beyotime Biotechnology, Inc. (Shanghai, China). The RPMI-1640 medium and fetal bovine serum (FBS) were purchased from Gibco BRL (Gaithersburg, MD, USA). The PRL was purchased from ProSpec (Hamada St., Rehovot, Israel). Polyclonal antibodies against β-casein, adipocyte differentiation-related protein (ADRP), E74-like factor 5 (Elf5), phospho-Akt_Ser473_ (p-Akt_Ser473_), Akt, and PRL receptor (PRLR) were bought from Cell Signaling Technology (Danvers, MA, USA). Secondary antibodies against β-actin and PI3K were bought from Bioss Biotechnology (Beijing, China). The polyclonal antibody against phospho-PI3K_Tyr508_ (p-PI3K_Tyr508_) was bought from Santa Cruz Biotechnology (Dallas, TX, USA). The related secondary antibodies were obtained from Bioworld Technology (St. Louis Park, MN, USA).

### 2.2. Cell Culture and Treatment

HC11 mouse mammary epithelial cells were maintained as previously described [18]. To induce cell differentiation, HC11 cells were cultured to reach 70%–80% confluence, and then incubated in medium deprived of epidermal growth factor for 24 h. Next, the cells were subjected to the following treatments in 12-well plates for four days: (1) control treatment: DIP (10 ng/mL dexamethasone, 5 μg/mL insulin, 5 μg/mL PRL), (2) LA treatment: LA (100 μM/mL) + DIP, and (3) LA treatment + 100 nM/mL WT. The cell culture supernatants were collected to determine the triglyceride (TG) secretions using a commercial kit (Nanjing Jiancheng Bioengineering Institute, Nanjing, Jiangshu, China). Additionally, all cells were collected for quantitative reverse-transcription (qRT-)PCR and western blot analysis. 

### 2.3. Animals and In Vivo Study

Twenty lactating mice were randomly divided into two groups (after delivery): a control group, which was fed with a control diet (AIN 93G; Guangdong Medical Laboratory Animal Center) (Table 1), and the LA group (control diet + 1% (W/W) LA, 1% LA rather than an equal amount of soybean oil). The calorie contents in the control and LA diets were the same. The mice were fed with these diets for 7 days, and the feed intake per day was recorded after delivery. On day 7 of lactation, the body weights of the mother and offspring were recorded. After being anaesthetized with carbon dioxide, the mice were collected and incubated at 37 °C for 1 h. The blood was then centrifuged at 2000 rpm for 20 min, and the serum was collected and stored at −20 °C until analysis. The collected serum was used to evaluate the PRL secretions with a commercial kit (Nanjing Jiancheng Bioengineering Institute, Nanjing, Jiangsu, China) in accordance with the instructions of the manufacturer. The fourth pair of mammary glands was fixed and used as a hematoxylin and eosin (H&E) staining material. The remaining mammary glands were frozen in liquid nitrogen and stored at −80 °C until analysis.

### 2.4. H&E Staining

H&E staining of the mammary gland tissues was actualized as previously described [18]. Curtly, the left side of the fourth pair of mammary glands was paraffin-embedded, and 5-μm-thick sections were provided and stained with H&E. A Nikon Eclipse Ti-s microscope (10×; Nikon Instruments, Tokyo, Japan) was used on the image capture. The alveoli area in each section was calculated using ImageJ software. The numbers of alveoli were counted in each section.

### 2.5. Western Blot Analysis

Proteins used for the western blot were obtained from mammary gland tissues or induced-HC11 cells, and the analysis was conducted as previously described [22]. Primary antibodies were applied to β-actin (1:5000), β-casein (1:2000), ADRP (1:2000), Elf5 (1:2000), PRLR (1:2000), Akt (1:2000), p-Akt_Ser473_ (1:2000), PI3K (1:2000), and p-PI3K_Tyr508_ (1:500). The immunoreactive proteins in the membrane were scanned with a FlourChemM Imaging System (ProteinSimple, Santa Clara, CA, USA). Densitometry was conducted using ImageJ software, and the band densities were normalized to that of β-actin. 

### 2.6. qRT-PCR

The qRT-PCR was used to examine the mRNA expression levels according to acyl-coenzyme A dehydrogenase medium chain (*ACADM)*, fatty acid synthase (*FASN)*, fatty acid transport protein 4 (*FATP4)*, and peroxisome proliferators-activated receptor γ (*PPARγ)*. The total RNA was extracted from the mammary glands using TRIzol (Invitrogen, Carlsbad, CA, USA) according to the manufacturer’s protocol. The total RNA was extracted from the cells using an RNA extraction kit (Guangzhou Magen Biotechnology, Guangdong, China). The RNA quality was assessed by measuring the absorbance at 260 and 280 nm, and the RNA samples with A260:A280 > 1.8 were used for further experiments. Briefly, cDNA was synthesized from 2 μg of total RNA using M-MLV Reverse Transcriptase (Promega, Madison, WI, USA) and random primers of oligo-dT18 according to the manufacturer’s instructions. qPCR was performed for 20-μL reaction mixtures, including SYBR Green Real-time PCR Master Mix reagents (Toyobo, Osaka, Japan) and sense and antisense primers (200 nM each), on a Mx3005p instrument (Stratagene, La Jolla, CA, USA). Table 2 shows the primer sequences and PCR fragment lengths. The relative target gene expression was calculated as described previously [23]. β-actin was used for normalization.

### 2.7. Lipidomics Analysis

The blood samples were thawed on ice, centrifuged for 10 s, and then centrifuged at 12,000 rpm for 10 min at 4 °C. Fifty microliters of the sample were homogenized with 1 mL of a mixture of methanol, methyl tert-butyl ether, and the internal standard. The time of centrifugation was 2 min, after which 500 μL of water was added and the mixture was centrifuged again for 1 min and then at 12,000 rpm at 4 °C for 10 min. The supernatants were dried with nitrogen. We used 100 μL of the mobile phase B for powder dissolution. The powder was stored at −80 °C. Then, the sample was transferred into a sample vial for liquid chromatography-tandem mass spectrometry (LC-MS/MS) analysis.

The sample extracts were analyzed by LC-electrospray ionization-MS/MS (LC-ESI-MS/MS; Ultra Performance Liquid Chromatography (UPLC), Shim-pack UFLC SHIMADZU CBM A system, Kyoto, Japan; MS, QTRAP^®^ 6500 + System, Framingham, MA, USA). The analytical conditions were as follows: UPLC: column, Thermo C30 (2.6 μm, 2.1 × 100 mm); solvent system, A: acetonitrile/water (60/40 V, 0.04% acetic acid, and 5 mmol/L ammonium formate), B: acetonitrile/isopropanol (10/90 V, 0.04% acetic acid, and 5 mmol/L ammonium formate); gradient program, A/B (80:20 V/V) at 0 min, 50:50 V/V at 3 min, 35:65 V/V at 5 min, 25:75 V/V at 9 min, 10:90 V/V at 15.5 min; flow rate, 0.35 mL/min; temperature, 45 °C; and injection volume: 2 μL. The effluent was alternatively connected to an ESI-triple quadrupole-linear ion trap (QTRAP)-MS.

The linear ion trap and triple quadrupole scans were gained on a triple QTRAP^®^ 6500+ mass spectrometer allocated with an ESI Turbo Ion-Spray interface, worked in positive and negative ion modes, and controlled with Analyst 1.6.3 software (Sciex). The ESI source operation parameters were grouped as: ion source, turbo spray; source temperature, 550 °C; ion spray voltage, 5500 V; ion source gas I, gas II, curtain gas at 55, 60, and 25 psi; (the collision gas was medium). The instrument tuning and mass calibration were worked with 10 and 100 μmol/L polypropylene glycol solutions in triple quadrupole and linear ion trap modes. Triple quadrupole scans were allocated as multiple reaction monitoring (MRM) experiments with the collision gas (nitrogen) set to 5 psi. The declustering potential and collision energy for individual MRM transitions were worked for further optimization of these parameters. According to the metabolites eluted within this period, a specific set of MRM transitions was monitored for each period.

### 2.8. Statistical Analysis

All data are expressed as the mean ± standard error of the mean (SEM). An individual animal was performed as an experimental unit and ten mice were tested in each group in the mouse feeding trial. There were three independent experiments conducted with at least three parallel measurements in each experiment. GraphPad Prism 7 (GraphPad Software, La Jolla, CA, USA) was used for the statistical analysis. The differences between the means were determined using Student’s t-test, and a confidence level of *p* < 0.05 was statistically significant.

## 3. Results

### 3.1. Dietary Supplementation of LA Improves Lactation Function in Breast-Feeding Mice

**Figure 1:**
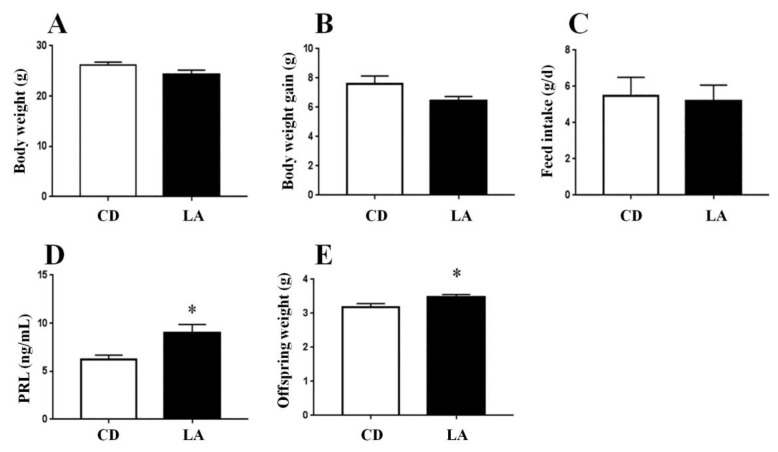
The effects of dietary supplementation of 1% lauric acid (LA) on lactating female mice and offspring mice. (**A**) The dietary 1% LA on the body weight of lactating mice. (**B**) The body weight gain of lactating mice. (**C**) The daily feed intake of lactating mice. (**D**) The content of PRL in the serum of lactating mice. (**E**) The average weight of offspring mice (n = 6). CD is control group. * *p* < 0.05 versus control group.

To examine the effect of LA feeding on lactating mice and offspring, we measured the body weight, body weight gain, feed intake, and serum PRL contents in breast-feeding mice, as well as the body weight of the offspring. We assessed the effect of dietary LA supplementation on lactation function by measuring the body weight of offspring mice and the serum PRL levels of breast-feeding mice. We observed no effect of LA supplementation on the body weight (Figure 1A), body weight gain (Figure 1B), or daily feed intake (Figure 1C) of breast-feeding mice, whereas LA supplementation significantly increased the body weight of offspring mice (Figure 1D), suggesting that LA improved the lactation function of breast-feeding mice. Accordingly, the serum levels of PRL, a hormone contributing to lactation function, were markedly elevated in the LA group (Figure 1E).

### 3.2. Dietary Supplementation of LA Promotes Mammary Gland Development in Breast-Feeding Mice

**Figure 2:**
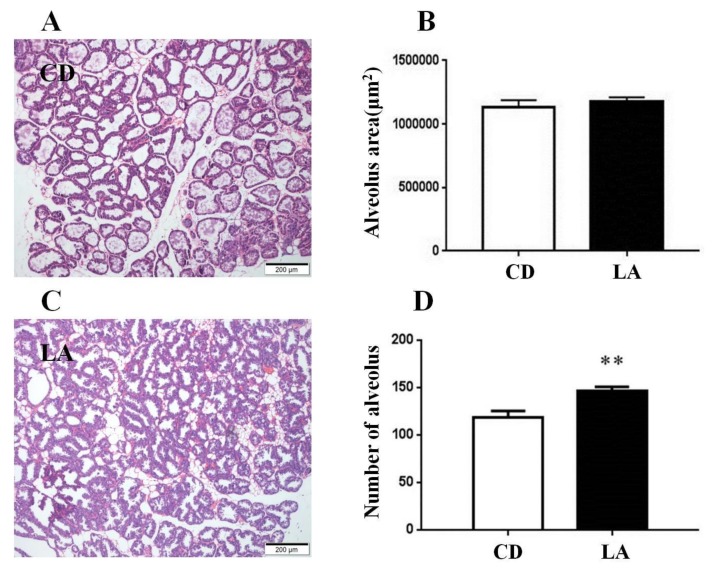
The results of H&E staining. (**A**,**C**) The fourth pair of mammary glands with control and 1% LA treated lactating mice, the scale bars are shown in the images. (**B**) The effect of dietary 1% LA on the alveolus area of mammary gland and the number of alveolus mammary glands (**D**) of lactating mice. CD is control group. ** *p* < 0.01 versus control group.

H&E staining (Figure 2A,C) revealed that there was no difference on the alveolus area of mammary glands between the two groups (Figure 2B). However, there was a significant increase in the number of alveoli in the mammary glands in the LA group compared with the control group (*p* < 0.01; Figure 2D).

### 3.3. Gene and Protein Expression in Mammary Glands of Lactating Mice

**Figure 3:**
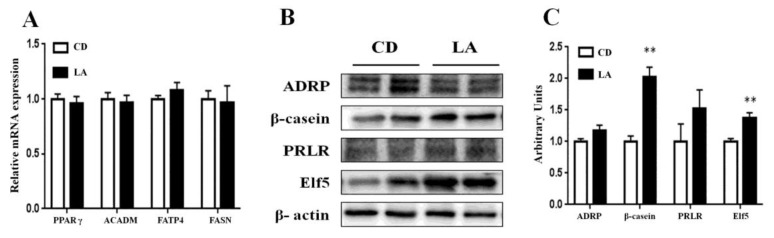
The effects of dietary supplementation of 1% LA on the relative gene expressions in the mammary glands of lactating mice. (**A**) The relative mRNA expression levels of *FASN, PPARγ, FATP4,* and *ACADM* in mammary glands (n = 4). (**B**) Western blot analysis of ADRP, β-casein, PRLR, and Elf5 in the mammary glands of lactating mice. β-actin was used as the loading control. (**C**) Mean ± SEM of immunoblotting bands of ADRP, β-casein, PRLR, Elf5; the intensities of the bands are expressed as arbitrary units (n = 4). CD is control group. ** *p* < 0.01 versus control group.

The effects of LA supplementation on the expression of genes and proteins related to mammary gland development and milk secretion were evaluated by qRT-PCR and western blot analyses of mammary gland tissues obtained from lactating mice. At the time points, the mRNA expression levels of fatty acid synthesis of *ACADM*, *FASN*, *FATP4*, and *PPARγ*, which are related to fatty acid synthesis remained unchanged (Figure 3A). As shown in Figure 3B,C, the protein expression levels of β-casein and Elf5 were significantly increased (*p* < 0.01), whereas the levels of ADRP, PRLR were not affected by LA supplementation.

### 3.4. TG Contents in Mammary Epithelial Cells and Cell Supernatants

**Figure 4:**
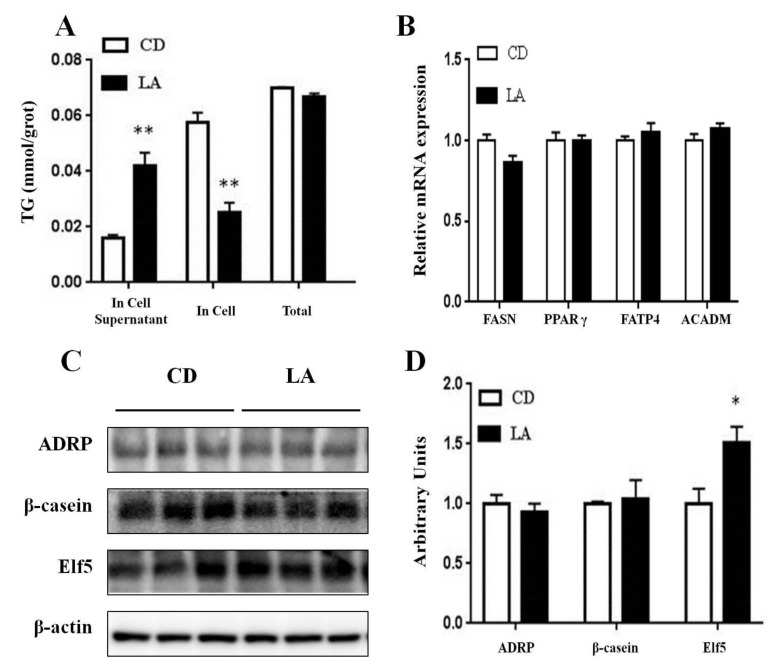
The effects of 100 μM LA on the content of TG and relative gene expressions in HC11 cells by DIP-induction. (**A**) The content of TG in HC11 cells and in the cell supernatant. (**B**) The relative mRNA expression level of *FASN, PPARγ, FATP4,* and *ACADM* in HC11 cells by DIP-induction (n = 4). (**C**) Western blot analysis of ADRP, β-casein, and Elf5 in HC11 cells by DIP- induction. β-actin was used as the loading control. (**D**) Mean ± SEM of immunoblotting bands of ADRP, β-casein Elf5, the intensities of the bands are shown as arbitrary units (n = 4). CD is control group. * *p* < 0.05 versus control group.

We detected the TG contents in HC11 cells and cell supernatants to determine the effect of LA on cellular fatty acid synthesis and secretion. As shown in Figure 4A, the TG content in HC11 cells was significantly decreased by LA (*p* < 0.01), whereas, the TG content in cell supernatants was significantly increased (*p* < 0.01). The total TG content in cells and supernatants, showed no significant difference between the two groups.

### 3.5. Gene and Protein Expression Related to Milk-fat Synthesis

Next, we detected the relative expression levels of *ACADM*, *FASN*, *FATP4*, and *PPARγ* in HC11 cells using qRT-PCR. As shown in Figure 4B, no differences were shown between the two groups. We observed no difference between the β-casein or ADRP protein expression between the two groups; however, interestingly, LA significantly increased the Elf5 protein expression in HC11 cells (*p* < 0.05; Figure 4C,D).

### 3.6. Inhibition of PI3K/Akt Blocked the Promotion of Differentiated HC11 Development by LA

**Figure 5:**
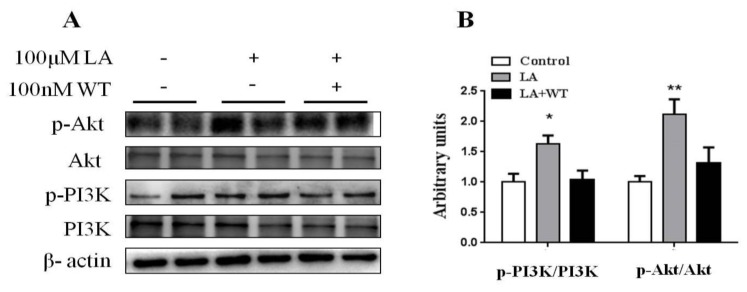
Inhibition of PI3K/Akt blocked the promotion of differentiated HC11 development by LA. (**A**) Western blot analysis of p-Akt, Akt, p-PI3K, PI3K in HC11 cell by DIP-induction. β-actin was used as loading control. (**B**) Mean ± SEM of the immunoblot bands of p-Akt/Akt, p-PI3K/PI3K, the intensities of the bands are expressed as arbitrary units (n = 4). * *p* < 0.05 versus control group. ** *p* < 0.01 versus control group.

We further verified the role of the PI3K/Akt signaling pathway in LA-stimulated cell development of differentiated HC11. In this study, WT was used to inhibit the PI3K activation and thus prevent Akt activation as a potent and selective inhibitor of PI3K. We found that the significant increases in the p-PI3K/PI3K and p-Akt/Akt ratios in response to 100 μM LA were reversed by 100 nM WT (Figure 5A,B). These results suggested that LA activated the PI3K/Akt signaling pathway in differentiated HC11.

### 3.7. Screening of Differential Lipid Metabolites in Serum of LA-Treated Lactating Mice

As the average weight of the offspring, and the protein expression of β-casein and Elf5 were significantly increased in the LA group (Figure 1E, Figure 3B,C), we investigated the effect of dietary LA on the blood lipid metabolism in lactating mice by screening for differential metabolites by super-high performance liquid chromatography-mass spectrum in series (UPLC-MS). As shown in Table 3, nine metabolites were significantly up-regulated by LA supplementation (e.g., the *sn*-2 position is C12, and the *sn*-1 position is C18 of TG). Additionally, 18 metabolites were significantly down-regulated upon LA supplementation, including eicosanoid, phosphatidyl ethanolamine, lysophosphatidyl ethanolamine, and phosphatidyl choline, and the *sn*-2 position in C14 of TG.

## 4. Discussion

The mice mammary gland development during lactation is influenced by PRL, progesterone, growth hormone, and insulin-like growth factor-1 [2]. We found that LA treatment significantly increased the PRL content of lactating mice serum. In previous studies, the addition of fatty acid to the diet of female mice led to significant increases in the average weight of the offspring [24,25]. This is similar to the results of our recent studies in lactating mice. The average weight of the offspring was significantly increased by dietary LA in lactating mice. We also found that the number of mammary gland alveoli in the LA group raised significantly more than that in the control group. The production performance reported in dairy cows was not related to the changes in fatty-acid gene expression in the mammary glands [26]. In this study, there were no significant differences in the mRNA expression of *ACADM*, *FASN*, *FATP4*, and *PPARγ* in the mammary glands.

Elf5 plays an important role in the proliferation and differentiation of mammary alveoli during pregnancy and lactation [27]. It is considered as one member of the Ets (E twenty-six) -domain transcription factor family. The expression of β-casein, WAP (whey acidic protein), and CEL (carboxyl ester lipase), which encode the milk proteins, are all regulated, either directly or indirectly by Elf5 [28]. Our results showed that the level of Elf5 protein expression was up-regulated with LA treatment in the mammary glands. In our study, LA could increase the protein expression level of p-mTOR/mTOR (mammalian target of rapamycin) in induced HC11 cells (not covered in the manuscript). This confirmed that mTOR is an important regulator of milk protein synthesis [29,30]. The LA supplement increased the β-casein protein expression in the mammary glands. Nutrients may promote β-casein synthesis by the mTOR route in BMEC (bovine mammary epithelial cells) [31]. 

The mTORC1 pathway is considered the main signal pathway for cell proliferation and protein synthesis [32,33]. We supposed that the increase of β-casein might be caused by the activation of mTORC1. ADRP has important effects on milk fat formation and secretion in mammary cells. ADRP mRNA and protein levels in the mammary glands of lactating mice are 30-fold higher than those in the mammary glands of non-lactating mice [34]. Previous studies have implicated ADRP as a physiological regulator of milk lipid production [35]. In our study, the ADRP expression was slightly, but not significantly, increased in the mammary glands of LA-supplemented mice. Thus, dietary supplementation of LA may promote mammary gland development by promoting the proliferation of mammary gland alveoli, promoting PRL release in lactating mice, and improving the growth performance of offspring.

We examined the effect of LA on lipid metabolites in the blood of lactating mice by lipidomics analysis. In total, 27 metabolites were differentially expressed upon dietary LA supplementation, among which, nine metabolites were up-regulated. The *sn*-2 position in C12 and *sn*-1 position in C18 of TG showed the strongest up-regulation, specifically sphingomyelin. This cell membrane component participates in physiological processes, such as cell growth, development, and proliferation [36,37]. Therefore, LA may up-regulate sphingomyelin in the blood to promote the development of mammary gland cells in female mice and, thus, improve the lactation performance.

The activation of the PI3K/Akt signaling pathway is coupled with regulating the proliferation of various cells [38,39,40]. The PI3K/Akt signaling pathway has effects on regulating mammary gland development [41,42]. There are some studies that show that the activation of the PI3K/Akt signaling pathway may enhance mammogenesis and lactogensis [43,44]. According to the previous studies, we found that LA could activate the PI3K/Akt signaling pathway during HC11 differentiation. WT, as an inhibitor of the PI3K/Akt signaling pathway, reversed the LA-induced stimulation of HC11 differentiation. These results indicated that LA stimulated HC11 differentiation by activating the PI3K/Akt signaling pathway. 

It was demonstrated that LA can stimulate HC11 proliferation and mammary ductal growth related to the activation of the PI3K/Akt signaling pathway through GPCR (G protein-coupled receptors) [18]. We speculated that LA might also activate the PI3K/Akt signaling pathway through GPCR. PRL plays its physiological role by activating signaling pathways related to the PI3K/Akt signaling pathway [44,45]. In our study, dietary LA markedly elevated the serum levels of PRL, which may activate the PI3K/Akt signaling pathway in lactating mice. in vitro, LA treatment increased TG release and decreased the cellular TG content; however, there was no significant difference in the total TG content. Furthermore, there were no significant differences in the expression of genes related to lipid synthesis. Thus, LA may promote TG secretion to the extracellular environment.

## 5. Conclusions

Our results suggest that LA promoted HC11 differentiation and mammary gland development in lactating mice through activating the intracellular PI3K/Akt signaling pathway and increasing the contents of sphingomyelin and other metabolites with the *sn*-2 position of C12 and *sn*-1 position of C18 in TG to improve the growth performance of the offspring. Thus, dietary supplementation of LA during lactation could promote the lactation function of mice, which might be associated with the enhancement of mammary gland development and the alternation of serum lipid metabolites.

## Figures and Tables

**Table 1:** The composition of the control diet (AIN 93G).

Ingredient	gm	kcal
Casein, 30 Mesh	200	800
L-Cystine	3	12
Corn Starch	397.5	1590
Maltodextrin	132	528
Sucrose	100	400
Cellulose	50	0
Soybean Oil	70	630
Mineral Mix #210025	35	0
Vitamin Mix #310025	10	40
Choline Bitartrate	2.5	0
Total	1000	4000
	gm%	kcal%
Protein	18.7	19.3
Carbohydrate	64.7	64
Fat	7	16.7
kcal/gm	4	

**Table 2:** The primer sequences used for quantitative reverse-transcription (qRT-)PCR.

Gene	Primer Sequences (5′–3′)	Amplification Length
*ACADM*	F: GGCCAGAAGATGTGGATAAC R: GTCGGCTTCCACAATGAA	123 bp
*FASN*	F: GGAGGTGGTGATAGCCGGTAT R: TGGGTAATCCATAGAGCCCAG	140 bp
*FATP4*	F:TCTGTTCTGATTCGTGTTCG R:CAGCATATACCACTACTGG	137bp
*PPARγ*	F: GGAAGACCACTCGCATTCCTT R: GTAATCAGCAACCATTGGGTCA	121 bp
β-actin	F: GGTCATCACTATTGGCAACGAG R: GAGGTCTTTACGGATGTCAACG	142 bp

**Table 3:** The screening results of differential metabolites.

Index	Compounds	Class	Type
LIPID-N-0006	9,10-EpOME	Eicosanoid	down
LIPID-N-0407	PE (18:2/16:0)	PE (Phosphatidyl ethanolamine)	down
LIPID-N-0410	PE (18:0/18:2)	PE	down
LIPID-N-0525	PE (22:6/16:0)	PE	down
LIPID-P-0272	LPE (0:0/22:1)	LPE (Lysophosphatidyl ethanolamine)	down
LIPID-P-0281	LPE (0:0/20:5)	LPE	down
LIPID-P-0528	PC(O-16:0/14:2)	PC (Phosphatidyl choline)	down
LIPID-P-0749	TG (14:0/16:0/16:0)	TG (Triglyceride)	down
LIPID-P-0792	TG (14:0/18:0/18:1)	TG	down
LIPID-P-0804	TG (18:0/18:0/18:1)	TG	down
LIPID-P-0834	TG (14:1/14:1/16:0)	TG	down
LIPID-P-0863	TG (14:0/18:2/22:0)	TG	down
LIPID-P-0894	TG (14:1/14:1/18:1)	TG	down
LIPID-P-0901	TG (14:0/16:1/18:2)	TG	down
LIPID-P-0902	TG (14:0/16:0/18:3)	TG	down
LIPID-P-0969	TG (14:1/14:1/18:2)	TG	down
LIPID-P-1036	TG (14:0/18:2/18:3)	TG	down
LIPID-P-1178	TG (14:0/20:5/22:4)	TG	down
LIPID-P-0716	SM(d18:0/12:0)	SM (Sphingomyelin)	up
LIPID-P-0724	SM(d18:1/12:0)	SM	up
LIPID-P-0833	TG (12:0/12:0/18:2)	TG	up
LIPID-P-0838	TG (14:0/14:0/18:2)	TG	up
LIPID-P-0839	TG (14:1/16:0/16:1)	TG	up
LIPID-P-0896	TG (12:0/16:0/18:3)	TG	up
LIPID-P-0970	TG (12:0/18:2/18:2)	TG	up
LIPID-P-0972	TG (12:0/18:1/18:3)	TG	up
LIPID-P-1031	TG (14:1/16:1/18:3)	TG	up
